# Successful treatment of severe arterial hypotension and anuria in a preterm infant with renal tubular dysgenesis– a case report

**DOI:** 10.1186/s40748-018-0095-z

**Published:** 2018-12-20

**Authors:** Katharina Ruf, Johannes Wirbelauer, Antje Beissert, Eric Frieauff

**Affiliations:** University Children’s Hospital Würzburg, Josef-Schneider-Straße 2, 97080 Würzburg, Germany

**Keywords:** Potter sequence, Oligohydramnios sequence, Renal tubular dysgenesis, Arterial hypotension, Vasopressin, Respiratory distress, Anuria, Preterm, Dry lung syndrome, Neonatal renal failure

## Abstract

**Background:**

Oligohydramnios sequence can be caused by renal tubular dysgenesis (RTD), a rare condition resulting in pulmonary and renal morbidity. Besides typical features of Potter-sequence, the infants present with severe arterial hypotension and anuria as main symptoms. Establishing an adequate arterial blood pressure and sufficient renal perfusion is crucial for the survival of these infants.

**Case presentation:**

We describe a male preterm infant of 34 + 0 weeks of gestation. Prenatally oligohydramnios of unknown cause was detected. After uneventful delivery and good adaptation the infant developed respiratory distress due to a spontaneous right-sided pneumothorax and required thoracocentesis and placement of a chest tube; he showed no major respiratory concerns thereafter and needed only minimal ventilatory support. Echocardiography revealed no abnormalities, especially no pulmonary hypertension. However, he suffered from severe arterial hypotension and anuria refractory to catecholamine therapy (dobutamine, epinephrine and noradrenaline). After 36 h of life, vasopressin therapy was initiated resulting in an almost immediate stabilization of arterial blood pressure and subsequent onset of diuresis. Therapy with vasopressin was necessary for three weeks to maintain adequate arterial blood pressure levels and diuresis. Sepsis and adrenal insufficiency were ruled out as inflammation markers, microbiological tests and cortisol level were normal. At two weeks of age, our patient developed electrolyte disturbances which were successfully treated with fludrocortisone. He did not need renal replacement therapy. Genetic analyses revealed a novel compound hyterozygous mutation of RTD. Now 17 months of age, the patient is in clinically stable condition with treatment of fludrocortisone and sodium bicarbonate. He suffers from stage 2 chronic kidney disease; blood pressure, motor and cognitive development are normal.

**Conclusions:**

RTD is a rare cause of oligohydramnios sequence. Next to pulmonary hypoplasia, severe arterial hypotension is responsible for poor survival. We present the only second surviving infant with RTD, who did not require renal replacement therapy during the neonatal period. It can be speculated whether the use of vasopressin prevents renal replacement therapy as vasopressin increases urinary output by improving renal blood flow.

## Background

Oligohydramnios sequence remains a challenge for the neonatologist as most affected neonates present with considerable pulmonary morbidity and arterial hypotension. Renal tubular dysgenesis (RTD) is a rare condition resulting in oligohydramnios sequence with refractory arterial hypotension significantly complicating the course of treatment. Diagnosis of RTD can be challenging due to the lack of concrete findings on renal ultrasound. Managing arterial hypotension and concomitant anuria is crucial for survival. We report a preterm infant with refractory hypotension and anuria which was successfully treated with vasopressin.

## Case presentation

### Perinatal and neonatal outcome

The male infant was born to a 23 year-old primi-gravida mother. Pregnancy was uneventful, fetal urinary tract appeared normal on ultrasound, the mother’s medical history was negative for any medication as was family history for renal or cardiovascular disease. While amniotic fluid volume seemed normal on routine ultrasound examinations, anhydramnios of unknown origin was observed at 32 weeks of gestation. No signs of tear or leak in the amniotic membrane were detected. Anhydramnios and pathological umbilical blood flow led to Caesarean section at 34 + 0 weeks of gestation. Birthweight was 2515 g (66th percentile), head circumference 31 cm (24th percentile), APGAR scores 9 at 5′ and 10 at 10 min, umbilical cord artery pH 7.35. The anterior fontanelle was wide and the infant showed features of Potter-sequence with contractures of wrist and ankle joints as well as epicanthus. He also presented with distinct general edema.

**Fig. 1:**
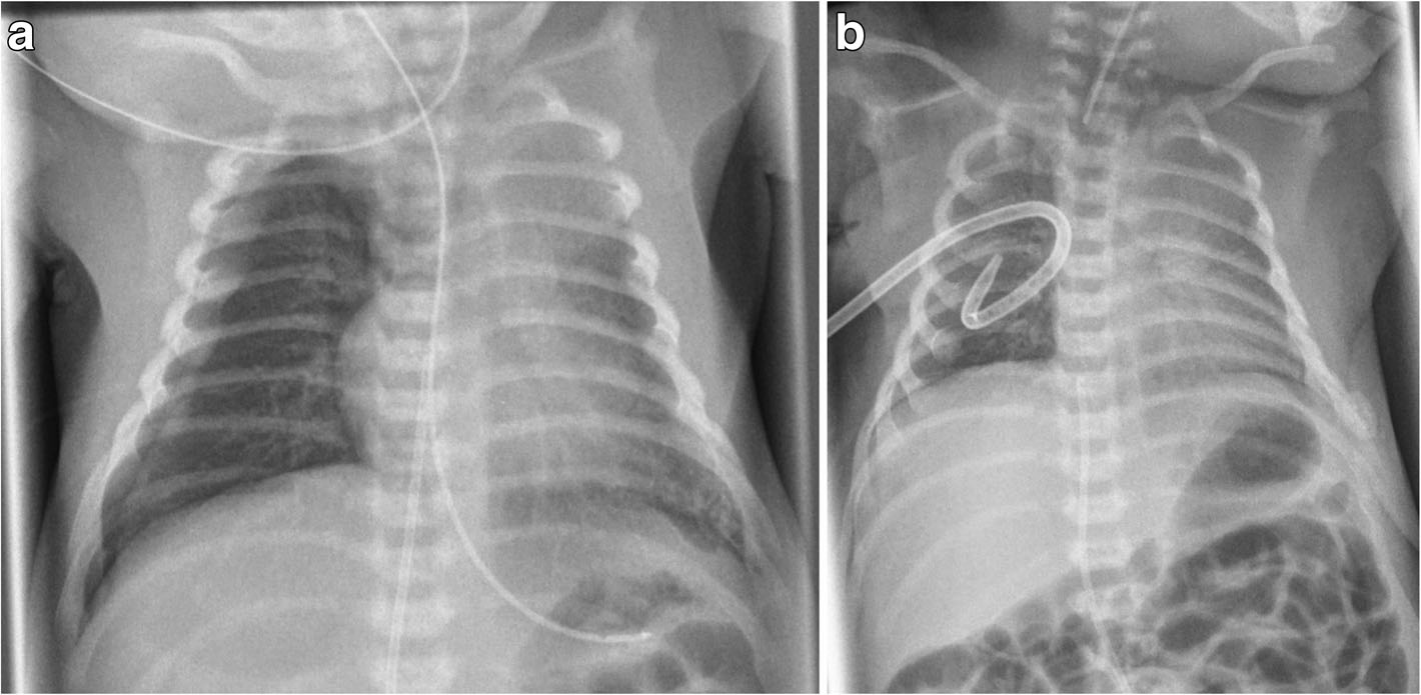
Chest X-rays on the first day of life. **a** spontaneous right-sided pneumothorax **b**) after intubation and chest tube insertion

After good postnatal adaptation, the infant needed mechanical ventilation due to respiratory distress caused by a spontaneous right-sided pneumothorax at the age of 1 h. He quickly stabilized after nasotracheal intubation and placement of a chest tube. Neither signs of pulmonary hypoplasia nor pulmonary hypertension were evident from chest x-ray or echocardiography (see Fig. 1a and b). Pre- and postductal oxygen saturation monitoring did not show any significant difference, ventilatory support was minimal (SIMV-mode, PIP 13 mbar, FiO2 0,25) and the patient had no signs of surfactant deficiency.

During the first day of life, he developed severe arterial hypotension (mean arterial blood pressure around 30 mmHg, below 10th percentile [1]) and consecutive renal failure and anuria (no urinary output for 36 h, serum creatinine 1,98 mg/dl, blood urea nitrogen (BUN) 120 mg/dl) which poorly responded to fluid boluses, catecholamine therapy (dobutamine, noradrenaline and epinephrine), furosemide and hydrocortisone therapy. Sepsis, cystic kidney disease and connatal nephrotic syndrome were ruled out. The cortisol level was normal. Echocardiography revealed good biventricular function without signs of pulmonary hypertension.

**Fig. 2:**
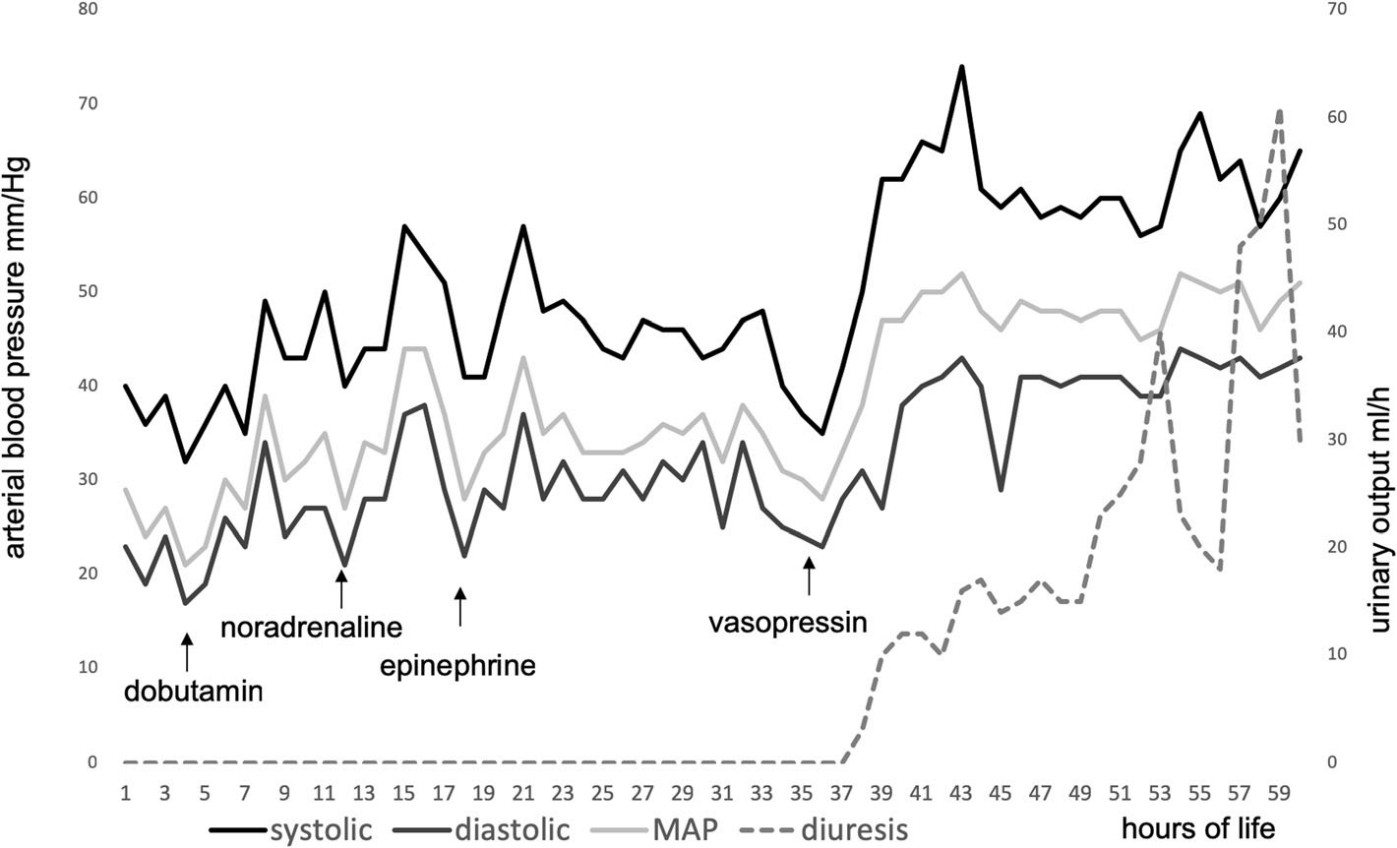
Blood pressure and diuresis during the first three days of life. This figure displays changes in blood pressure (systolic, diastolic and mean arterial pressure) as well as rate of diuresis in relation to the medication applied. See the immediate onset of diuresis after the initiation of vasopressin therapy

**Fig. 3:**
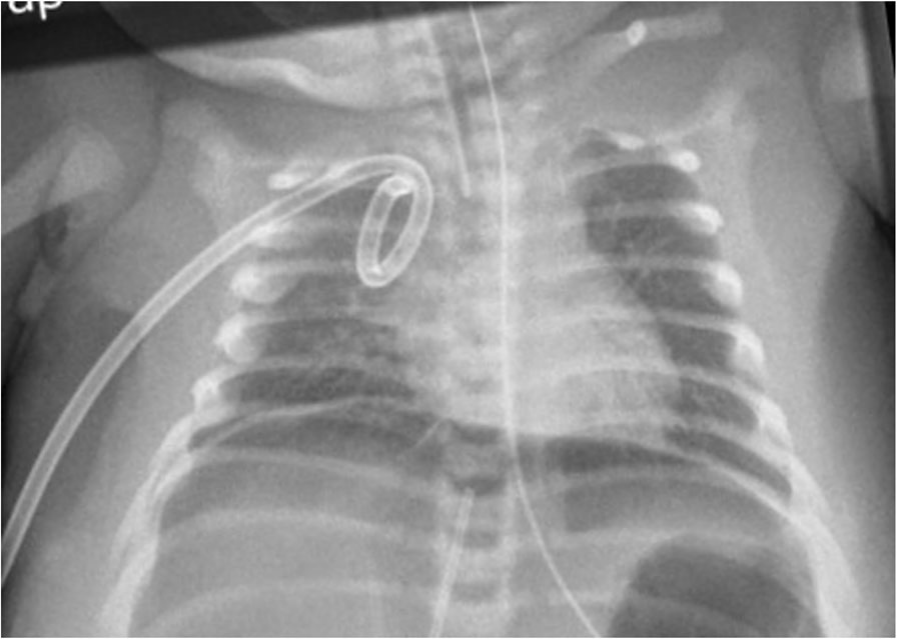
Chest X-ray depicting free abdominal air due to gastric perforation

After 36 h, vasopressin therapy was initiated with excellent response at doses of 0,001 IE/kg/min; blood pressure stabilized with consecutive onset of diuresis almost immediately after starting of vasopressin (see Fig. 2). Initially elevated creatinine and blood urea nitrogen normalized with the onset of diuresis. Weaning of vasopressin, however, was difficult due to rapid deterioration of blood pressure and urinary output and could not be discontinued for the next three weeks. Oligohydramnios, refractory arterial hypotension, renal failure with normal renal ultrasound was highly suggestive of renal tubular dysgenesis. On the fourth day of life, the patient suffered from a spontaneous gastric perforation, which was surgically treated without complications (see Fig. 3). On the 21st day of life, hyperkalemia needed to be treated with repetitive doses of furosemide. As renal tubular dysgenesis was suspected, endocrinological assessment was performed on day 12. It revealed an excessively high active renin concentration > 330 ng/l (normal 6.3 to 149 ng/l), a low concentration of ACE < 8 U/l (normal 8.3 to 21.4 U/l) and hypoaldosteronism (aldosterone < 37 ng/l, normal 73–425 ng/l). As this is a common finding in RTD, we established a fludrocortisone therapy resulting in stable electrolytes and bicarbonate. The infant required a gastric tube due to poor feeding until the 6*th* week of life. He was discharged at the age of 7 weeks.

Mutational analysis of the ACE gene showed a novel compound hyterozygous mutation. C.5303 + 1G > A has been described before and impairs splicing of pre-mRNA of ACE [2]. The other mutation was c.1487G > A and has not been reported before in RTD. Therefore, we present a patient with a novel compound hyterozygous constellation of RTD.

### Long term outcome

The patient is now 17 months of age and has been re-admitted several times for dehydration due to poor feeding in times of respiratory infection. Whenever fludrocortisone therapy was discontinued due to non-compliance, the patient developed hyperkalemia and rise of creatine levels. Currently he is suffering from stage 2 chronic renal disease with a GFR of 60 ml/min/1,73m^2^ (normal > 90 ml/min/1,73m^2^). Renal ultrasound shows increased echogenicity but no enlargement of the kidneys. With reestablished fludrocortisone therapy and sodium bicarbonate substitution electrolytes and blood urea nitrogen are normal, serum creatinine and cystatine C only slightly elevated (Creatinine 0,45 mg/dl, cystatine C 1,43 mg/l). Blood pressure is on the 50th percentile. Cognitive and psychomotor development are age-appropriate.

## Discussion

RTD is a rare disorder, characterized by prematurity, oligo- or anhydramnios, severe arterial hypotension and neonatal renal failure. It is a hyterozygous inherited autosomal-recessive disease with over 50 reported mutations [2]. It affects angiotensinogen, angiotensinogen receptor 1, renin or angiotensin converting enzyme genes in the Renin angiotensin system (RAAS) without a predictive genotype-phenotype correlation so far. Similar clinical findings have been observed in children exposed to RAAS blockage during pregnancy [3], highlighting the importance of RAAS for kidney development. So far, about 150 cases of RTD have been published [2], however, only about 10 long-term survivors have been reported [2, 4]; in most cases RTD is fatal either in utero or shortly after birth [5]. Differentiated proximal tubules are significantly reduced or even absent [5] on histological examinations of the patients’ kidneys. Prenatal signs of RTD are oligo- or anhydramnios and lack of concrete findings in prenatal urinary tract ultrasound. After birth, hallmark signs are a varying degree of Potter sequence with facial abnormalities and pulmonary hypoplasia, hypocalvaria and contractures of joints [2]. Patients present with severe, therapy refractory arterial hypotension, anuria and usually need respiratory support [5].

Hypotension and anuria amongst reported survivors were refractory to the usual treatment of fluid boluses, furosemide, catecholamine therapy and hydrocortisone treatment. Nearly all patients required peritoneal dialysis for the first days or weeks of life until diuresis was established. In two reported cases the continuous infusion of Fresh Frozen Plasma helped to increase blood pressure but peritoneal dialysis was still necessary [6, 7]. Only one child is reported so far that did not need peritoneal dialysis. Richer et al. report a preterm infant (26 + 5 gestational age) developing adequate blood pressure and diuresis when started on continuous vasopressin infusion on day 8 of life [8]. Our patient is only the second one described without renal replacement therapy in the neonatal period and also the second one having received vasopressin.

The application of intravenous vasopressin increases arterial blood pressure by a direct vasoconstrictive effect via V1 receptors and a local inhibition of nitric oxide production. At the same time, renal blood flow is enhanced possibly through selective efferent arteriolar constriction and nitric oxide mediated afferent arteriolar vasodilatation [9]. Some studies have found vasopressin helpful in catecholamine refractory arterial hypotension in septic and cardiac shock although its role is not yet clear [10]. In these studies, vasopressin significantly raised mean arterial blood pressure and urinary output [9, 11].

In all reported survivors of RTD, blood pressure and diuresis normalized, hypoaldosteronism though, persisted in most cases, rendering a fludrocortisone therapy necessary [2, 4, 6–8].

Our patient suffered from spontaneous gastric perforation. The mechanism is unclear, a hypoperfusion of the intestines and some intraluminal pressure for example due to the gastric tube may be a likely explanation. Few reports exist on gastric perforation through nasogastric tubes in preterm infants as most perforations are seen in the esophagus [12]. The patient reported by Kim et al. developed an ileac perforation in the neonatal period [4] which may be related to arterial hypotension in RTD, the patient reported by Richer et al. suffered from necrotizing enterocolitis. Whether this is related to RTD or extreme prematurity remains uncertain [8]. Since the number of survivors reported is small, it is difficult to assess whether a certain percentage of these patients suffers from intestinal perforations; however, due to hypoperfusion of the intestines a causative relation seems possible. Whether intestinal hypoperfusion is the result of underlying disease or impaired microcirculation due to the use of vasopressors remains unclear. Also, the administration of hydrocortisone has been associated with spontaneous intestinal perforations in the past [13]. So far, these have not been described in secondary RTD caused by exposure to RAAS blockers during pregnancy [3]. In patients who developed intestinal perforations, peritoneal dialysis is not an option; all reported patients were born preterm and of very low birthweight making also hemodialysis impossible.

As the patient described by Kim et al. [4], the use of vasopressin may have prevented renal replacement therapy in our patient by establishing sufficient renal perfusion and consecutively urinary output.

In the few survivors reported, chronic or end-stage renal disease is present [2], in one case renal transplantation at the age of 4 was performed [14], another patient needed peritoneal dialysis [7]. However, as stated by the authors, all patients show normal cognitive development.

## Conclusions

RTD is a rare, severe, often fatal disease and should be considered in infants with oligohydramnios sequence without sonographic renal abnormalities suffering from severe arterial hypotension. Respiratory failure and severe hypotension with anuria are the major challenges in the first days of life. Early treatment with vasopressin prevented peritoneal dialysis in the two cases known and may be the vasopressor of choice in this disease. It seems to be the only agent for establishing sufficient renal perfusion. Whether vasopressin is the key to treating arterial hypotension and preventing renal replacement therapy in patients with RTD needs to be further studied. If RTD is suspected in an infant and severe arterial hypotension persists despite high doses of catecholamines, the use of vasopressin may be considered a therapeutic option.

## Abbreviations

ACE: Angiotensin converting enzyme
BUN: Blood urea nitrogen
RAAS: Renin-angiotensin-aldosterone-system
RTD: Renal tubular dysgensis
